# Deep learning-based restoration of PET images from a dual-panel breast dedicated scanner^☆^

**DOI:** 10.1016/j.zemedi.2026.01.001

**Published:** 2026-01-14

**Authors:** Hamid Nezampour, Mehdi Amini, Amirhossein Sanaat, Habib Zaidi

**Affiliations:** a Department of Nuclear Physics, Faculty of Science, University of Mazandaran, P. O. Box 47415-416, Babolsar, Iran; b Division of Nuclear Medicine and Molecular Imaging, Geneva University Hospital, CH-1211 Geneva 4, Switzerland; c Department of Nuclear Medicine and Molecular Imaging, University of Groningen, University Medical Center Groningen 9700 RB Groningen, Netherlands; d Department of Nuclear Medicine, University of Southern Denmark, DK-500 Odense, Denmark; e University Research and Innovation Center, Óbuda University, Budapest, Hungary

**Keywords:** PET, Breast imaging, Image quality, Image restoration, Deep learning (DL)

## Abstract

Positron Emission Tomography (PET) is important for breast cancer diagnosis and monitoring, but high costs restrict access. Dual-panel scanners can reduce costs, though they typically produce lower quality images with quantitative bias compared to full-ring systems. In this study, we investigated the use of deep learning (DL) to address these limitations and improve image quality in a dedicated dual-panel breast PET scanner. Monte Carlo simulations were performed with the GATE toolkit to model both dual-panel and full-ring scanners. The dual-panel configuration included two detector heads separated by 21 cm, each consisting of 3×4 blocks of 13×13 crystals, while the full-ring system comprised 14 detector blocks in four rings with a 21 cm diameter. During acquisition, the dual panel system was rotated by 90 degrees (step and shoot, no data acquisition during motion) to increase angular sampling*.* Clinical data from 51 ^18^F-FDG breast PET/CT cases were used as activity and attenuation maps for the simulations. A SwinUNETR architecture was trained to synthesize full-ring–equivalent images from dual-panel data. Performance was evaluated with structural similarity index (SSIM), peak signal-to-noise ratio (PSNR), root mean square error (RMSE), and voxelwise correlation. The dual-panel and full-ring scanners achieved spatial resolutions of 3.2 mm and 1.6 mm, and sensitivities of 8.9 and 14.2 cps/kBq, respectively. Compared with dual-panel images, AI-enhanced outputs showed improvements of 2.65% in PSNR, 26.4% in SSIM, and 12.1% in RMSE. Voxelwise correlation increased markedly (R^2^ increased from 0.75 to 0.96). These findings highlight the potential of DL-based approaches to generate higher quality, artifact-reduced breast PET images, allowing cost-effective dual-panel systems to approach the performance of full-ring scanners.

## Introduction

Breast cancer (BC) is the most frequently diagnosed cancer among women, accounting for roughly one in eight of all cancer diagnoses. BC is the second-leading cause of cancer-related death worldwide after lung cancer [1], [2], [3]. BC diagnosis and treatment planning rely on accurate and detailed imaging data. Positron emission tomography (PET) provides valuable functional and metabolic information for diagnosis and staging of breast cancer, and consequent metastases. However, the high cost of whole-body PET scanners and their maintenance make their availability challenging for developing countries [4]. Furthermore, whole-body PET scanners are not optimized for breast scanning, as the large gantry reduces significantly spatial resolution and sensitivity for breast imaging.

To address these challenges, dedicated breast PET scanners have been proposed, potentially offering improved performance compared to whole-body PET scanners [5]. Several studies have shown improved performance of breast dedicated PET scanners in detecting BC, predicting prognosis, assessing response to therapy, and discriminating between benign and malignant lesions [6], [7], [8], [9]. Physically, this is a very challenging site owing to the high level of tracer uptake within the chest. Several PET systems, including dual-head, C-shaped, and dual arc-shaped configurations, have been proposed [10], [11], [12]. The Montreal group has done pioneering efforts in developing positron emission mammographs [13]. Since this early work, several designs of breast PET scanners have been developed, including a few commercial systems, such as the Oncovision MAMMI design [14]. However, PET scanners with partial-ring geometry have been suggested to reduce the cost and serve diverse imaging objectives [15]. The dual-head PET system enables online radiation therapy, unlike full-coverage PET scanner [16]. A dual-head planar PET scanner has been employed for breast cancer detection, showing promising results in terms of lesion detectability [17], [18]. Active-PET represents a conceptual design of a PET scanner equipped with movable detectors that can be positioned closer to the breast, thereby enhancing sensitivity and spatial resolution [16].

The partial-ring PET design lacks complete angular sampling, which might lead to missing or distorted information, high level of noise and appearance of artifacts during image reconstruction. This consequently adversely affects the accuracy and reliability of quantitative image analysis, resulting in limited attention towards partial-ring PET scanners [19]. To mitigate the issue of missing data, various approaches have been suggested, either in the projection domain or during the image reconstruction process. Prior studies utilized different interpolation methods to estimate the missing projection data [20], [21], [22]. Projection-domain-based approaches showed significant improvement in reducing streak artifacts. However, they may tend to produce overly smooth images [23]. On the other hand, advanced iterative image reconstruction methods, such as the penalized weighted least squares method [24], dictionary learning [25] and compressed learning [23] demonstrated the capability of reducing streak artifacts while preserving important image details effectively. However, it is worth noting that image reconstruction using these methods may be time-consuming. Additionally, optimizing data-dependent regularization parameters remains challenging.

In recent years, artificial intelligence (AI), particularly deep learning (DL) algorithms, has gained significant traction in medical imaging. DL-based methods have primarily focused on enhancing resolution and sensitivity, with the aim of improving the overall performance of PET scanners [26], [27], [28], [29], [30], [31]. Several recent studies have shown that DL-based methods could reduce streak artifacts caused by limited angular measurements [32], [33], [34], [35], [36].

The aim of leveraging DL techniques in partial-geometry coverage breast PET scanners is to recover the missing data, improve image resolution, enhance the visibility of small lesions, and reduce image artifacts. These advancements can lead to more accurate detection and characterization of breast lesions, aiding in early diagnosis and personalized treatment planning. Therefore, it is possible to reduce the scanner's detectors and design a cost-effective breast PET, while the image quality is preserved. It is important to acknowledge that while DL techniques hold great promise, there are still challenges to address, such as the requirement for large, annotated datasets, the risk of overfitting, and the interpretability of DL models.

In this study, we simulated a dedicated partial-PET scanner using GATE (Geant4 Application for Tomographic Emission) [37] and enhanced image quality through a DL models. We simulated a partial- and full-ring PET scanners with detector modules having similar physical characteristics. We compared the performance of these two scanners in terms of spatial resolution at 1 cm radial distance from the center of the field-of-view (CFOV), and sensitivity at the CFOV, and additional image quality metrics. A clinical dataset including 51 breast PET/CT images were used to generate activity and attenuation maps required as input for simulations. A Shifted Window + U-Net Transformer (SwinUNETR) model was trained to synthesize the full-ring from the partial-ring PET images. The hierarchical features of the SwinUNETR allow the model to capture both global context and fine-grained local details, which is critical for reconstructing PET images where missing angular information can blur small lesions. The UNETR-based decoder structure ensures that spatial information is preserved through skip connections, facilitating accurate recovery of lesion boundaries and subtle structures. Moreover, the modular and scalable design of SwinUNETR allows adaptation to datasets of different resolutions and sizes, which is important in medical imaging tasks with limited training data. These combined properties make SwinUNETR particularly well-suited for PET image quality enhancement compared to conventional CNN-based architectures [38]. The reconstructed PET images obtained from the full-ring PET scanner were considered as ground truth in this study. The ground truth images were compared with the initial dual-panel images and the quality enhanced images by DL.

## Materials and methods

### Monte Carlo simulation of the scanners

Monte Carlo simulations were performed using the open-source code GATE simulation package[39]. Both the dual-head and cylindrical PET scanners were implemented using the GATE toolkit. All simulations were performed using GATE version 8.2 (based on Geant4 version 10.5) with the EmStandardOpt3 physics list. The production cuts were set to 1 mm for all particle types.

#### Detector blocks configuration

As a baseline, the physical characteristic of the commercially available Biograph mCT scanner (Siemens Healthineers, Erlangen, Germany) were used for designing our scanner. Each detector block included a 13×13 Lutetium Oxyorthosilicate (LSO) scintillation crystal, with an inter-module spacing of 25 μm. The system operates with a coincidence time window of 4.1 ns and an energy window of 435–650 keV [40].

#### Full-ring PET scanner configuration

**Fig. 1:**
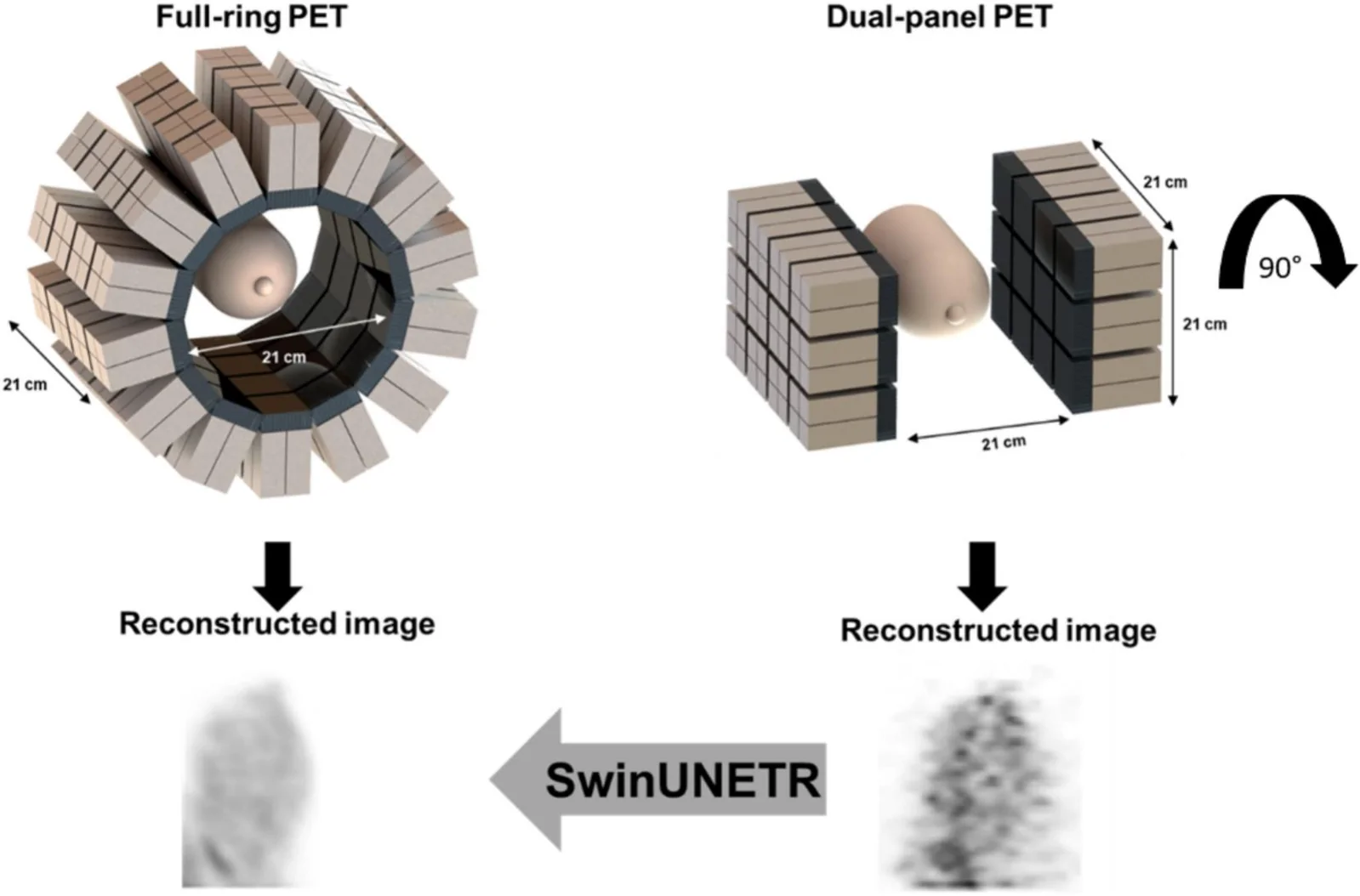
Illustration of the two breast PET scanners geometries including the full-ring (left) and dual-panel PET (right) designs showing the Swin UNETR deep learning model used to predict PET images corresponding to the full-ring geometry from the dual-panel PET images.

The full-ring breast PET scanner configuration was designed with 14×4 detector blocks arranged in a cylindrical configuration with 4 rings to cover a single breast. The transaxial FOV of this scanner is 21 cm (Fig. 1). The detector blocks in the Siemens Biograph mCT PET scanner are separated by 4 mm gaps between block rings.

#### Partial-ring PET scanner configuration

For the partial-breast PET configuration, each of the two planar detectors consisted of 3×4 detector blocks, positioned opposite to each other at a distance of 21 cm (Fig. 1). This configuration is specifically designed to optimize imaging of the breast. The acquisition was performed in a step and shoot manner using two discrete poses. The system acquired coincidences in the 0°-pose for the first half of the scan, then rotated to 90° and acquired coincidences for the second half. No data were recorded during the mechanical transition. The split between poses was based on equal acquisition time, consistent with the protocol used in the simulations.

The simulated PET scanners, apart from the detectors' size and arrangement, maintained consistent physical characteristics, including the time and energy windows, crystal material, dead-time, and time-of-flight (TOF) resolution, as the Biograph mCT scanner. More details regarding the simulation setup can be found in our previous works [41], [42]. Table 1 summarizes the physical characteristics of the designed scanner.

**Table 1:** Physical characteristics of the simulated detector blocks.

Parameter	Value
Axial FOV (mm)	210
Crystal material	LSO
Crystal length (mm)	20
Crystal pitch (mm)	2.7
Crystals per block	13×13
Blocks per module	43
Time resolution	527.5 ps
Coincidence time window	4.1 ns
Energy window	435-650 keV

### Validation of the simulation model

The simulation code of the Biograph mCT scanner was previously validated based on the NEMA standards reported in various studies [16], [40]. This code was changed by reducing the number of detector blocks and gantry diameter to simulate a full-ring and dual-panel breast PET scanner. A previously developed dedicated breast PET scanner has been reported in [43] .Although the scanner configurations were not completely similar, the spatial resolution and sensitivity were in the same range. The calculated metrics are summarized in Table 1.

To evaluate the spatial resolution of the dual-panel PET scanner more comprehensively, we assessed the spatial resolution and sensitivity at multiple radial positions from the CFOV. These position-dependent measurements are crucial in limited-angle geometries due to varying acceptance angles and resulting anisotropic resolution. The spatial resolution was determined in the radial (x), tangential (y), and axial (z) directions using a simulated point source of ^18^F placed in air. Simulations were performed with the point source located at radial offsets of 1 cm, 5 cm, and 10 cm from the CFOV and full width at half maximum (FWHM) values were extracted in all three spatial directions. Similarly, sensitivity was calculated using a line source placed along the axial direction at the same radial positions (0 cm, 5 cm, and 10 cm). Sensitivity was reported as counts per second per kilobecquerel (cps/kBq) by recording the total number of true coincidences.

We utilized the ordered subsets-expectation maximization (OSEM) reconstruction algorithm implemented within Customizable and Advanced Software for Tomographic Reconstruction CASToR [44]. We also assessed and compared our simulation results with experimental results obtained from dedicated breast PET scanners [45]. The reconstructed point source images were analyzed using the AMIDE software to directly calculate the FWHM values from line profiles.

### Image reconstruction

CASToR was employed to perform image reconstruction for cylindrical PET scanner geometries [44]. For the full-ring geometry, images were reconstructed using the standard clinical OSEM protocol with 5 iterations and 21 subsets to ensure comparability with routine practice. For the dual-panel configuration, reconstruction was performed with 10 iterations and 16 subsets, which provided the best balance between resolution recovery and noise amplification in the limited-angle setting. These reconstruction parameters were applied consistently for all corresponding simulations and analyses. Attenuation correction was applied by incorporating the attenuation maps derived from clinical PET/CT images into the reconstruction process. Scatter correction was not explicitly modeled in this study, given the simulation-based nature of the dataset and the primary focus on evaluating deep learning-based image enhancement.

CASToR assumes a fixed detector geometry for a given reconstruction, and the standard GATE to CASToR geometry conversion does not represent a detector pose that changes during acquisition. To model the adopted two-pose acquisition, we used a custom lookup table (LUT) containing crystal coordinates for both the 0° and 90° poses.

### Activity and attenuation maps for GATE simulation

Clinical PET and CT images were used as the activity and attenuation inputs for all simulations. The PET and CT volumes had a matrix size of 128 ×128 × 10 with an isotropic voxel size of 1 mm. The PET images, expressed in Bq/mL, were imported into GATE as voxelized activity sources. The CT images were converted into linear attenuation coefficients and used as the attenuation map for photon transport. The breast region was modeled directly from the CT-based material map using the corresponding GATE tissue definitions for soft tissue. All preprocessing steps, including image resampling and format conversion, were performed in MATLAB before importing the volumes into GATE. Voxels in the breast were classified using HU ranges consistent with fatty tissue (around −35 HU), glandular tissue (around +40 HU), low-density cystic lesions (about 13–27 HU), and higher-density solid masses (from 30 up to above 100 HU). This approach models the breast as a heterogeneous mixture of tissue types rather than a single water-equivalent material with density scaling. A bilinear Hounsfield Unit to density map relationship was used to convert CT numbers to the corresponding material densities.

### Clinical image simulation

The data used in this work were obtained from the QIN-BREAST public dataset (Vanderbilt University Medical Center and University of Chicago). They were acquired on a GE Discovery STE scanner (GE Healthcare, Waukesha, WI, USA). A low-mAs CT scan was acquired for attenuation correction of the emission data. The acquisition parameters for the transmission CT scan were as follows: the tube current was 80 mAs for a 70-kg patient and scaled accordingly for all patients, the tube voltage was 120 kVp, and the pitch was 1.675:1. The activity of FDG administered was approximately 370 MBq (10 mCi) for a 70-kg patient and scaled according to weight. FDG was administered intravenously via an antecubital vein contra-lateral to the affected breast. [46]. Accordingly, the number of detected events differs between the two scanners, with approximately ∼4.05 × 10^5^ true coincidence events for the dual-panel system and ∼1.87 × 10⁶ for the full-ring scanner.

The 51 ^18^F-FDG breast PET images, representing both normal and abnormal cases across various stages of breast cancer, were used as activity and attenuation maps for simulation. These images were reconstructed using an ordinary Poisson ordered OP-OSEM algorithm using CASToR [44]. For data processing, the images were cropped and adjusted using MATLAB code to prepare them for further analysis and integration into the dataset.

### Deep neural network architecture

We used Swin UNETR, which leverages the hierarchical features of Swin Transformer and the semantic segmentation capabilities of UNETR, resulting in a powerful model for tasks, such as image quality enhancement and image generation [47]. The Swin UNETR architecture offers a scalable and modular approach, making it adaptable to different datasets and domains within the field of computer vision [38], [48]. In this study, we implemented a modified version of the SwinUNETR architecture [49]. Specifically, we reduced the feature map size, adjusted the bottleneck depth, and modified the skip connections. These modifications were introduced to balance model performance with the limited dataset size and to ensure efficient training and generalization. By doing so, the architecture was adapted to better preserve fine structural details in PET images while maintaining computational efficiency*.* The utilization of Transformer-based models has gained significance in the field of computer vision. These models were first introduced in [50] with the objective of applying successful self-attention capabilities seen in natural language processing (NLP) to tasks related to images and vision. Our design included essential components like an encoder, a bottleneck, a decoder, and skip connections, all centered on the Swin-transformer (shifted windows) module. Image processing began by dividing input images into non-overlapping 4×4 blocks, which were linearly projected to generate sequences for network input. The encoder utilizes patch-merging blocks for signal down-sampling and Swin-transformer blocks for representation learning, creating a hierarchical representation. This representation structure mirrors the U-Net's U-shaped architecture, featuring a symmetric decoder layer comprising Swin-transformer layers and patch-expanding units. To ensure seamless signal transmission, skip connections are established between the encoder and decoder. At the core of the encoder, a bottleneck is formed, consisting of two consecutive Swin-transformer blocks. It is noteworthy that this bottleneck does not involve any up- or down-sampling operations and acts as an additional connection between the encoder and decoder components.

In the Swin UNETR architecture, several key components contribute to its effective performance in image restoration tasks. The Multi-head Self-Attention (MSA) mechanism enables the model to capture long-range dependencies and global contextual information within each local window. To enhance modeling efficiency and reduce computational overhead, the architecture incorporates Shifted Windows (SW), which allow for cross-window connections and improved representation learning across neighboring patches. Each layer also integrates Layer Normalization (LN) to stabilize training and normalize feature maps, ensuring consistent learning dynamics throughout the network. Within the attention mechanism, *W* denotes the window over which the attention operation is computed, enabling localized and efficient attention modeling. These integrated components collectively empower the Swin UNETR to accurately reconstruct high-quality PET images from limited-angle input data [38], [48].

(1) $${\hat{r}}^{l}=W\text{-}MSA\left(LN\left(r^{l-1}\right)\right)+r^{l-1}$$

(2) $$r^{l}=MLP\left(LN\left({\hat{r}}^{l}\right)\right)+{\hat{r}}^{l},$$

(3) $${\hat{r}}^{l+1}=SW\text{-}MSA\left(LN\left(r^{l}\right)\right)+r^{l},$$

(4) $$r^{l+1}=MLP\left(LN\left({\hat{r}}^{l+1}\right)\right)+{\hat{r}}^{l+1},$$

In this context ${\hat{r}}^{l}$ and $r^{l}$ stand for the outputs of the W-MSA and the Multi-Layer Perceptron (MLP) module of the $l^{th}$ block, respectively. The self-attention is computed as follows:

(5) $$Attention\left(q,k,v\right)=Softmax\left(\frac{qk^{T}}{\sqrt{d}}\right)v,$$

**Fig. 2:**
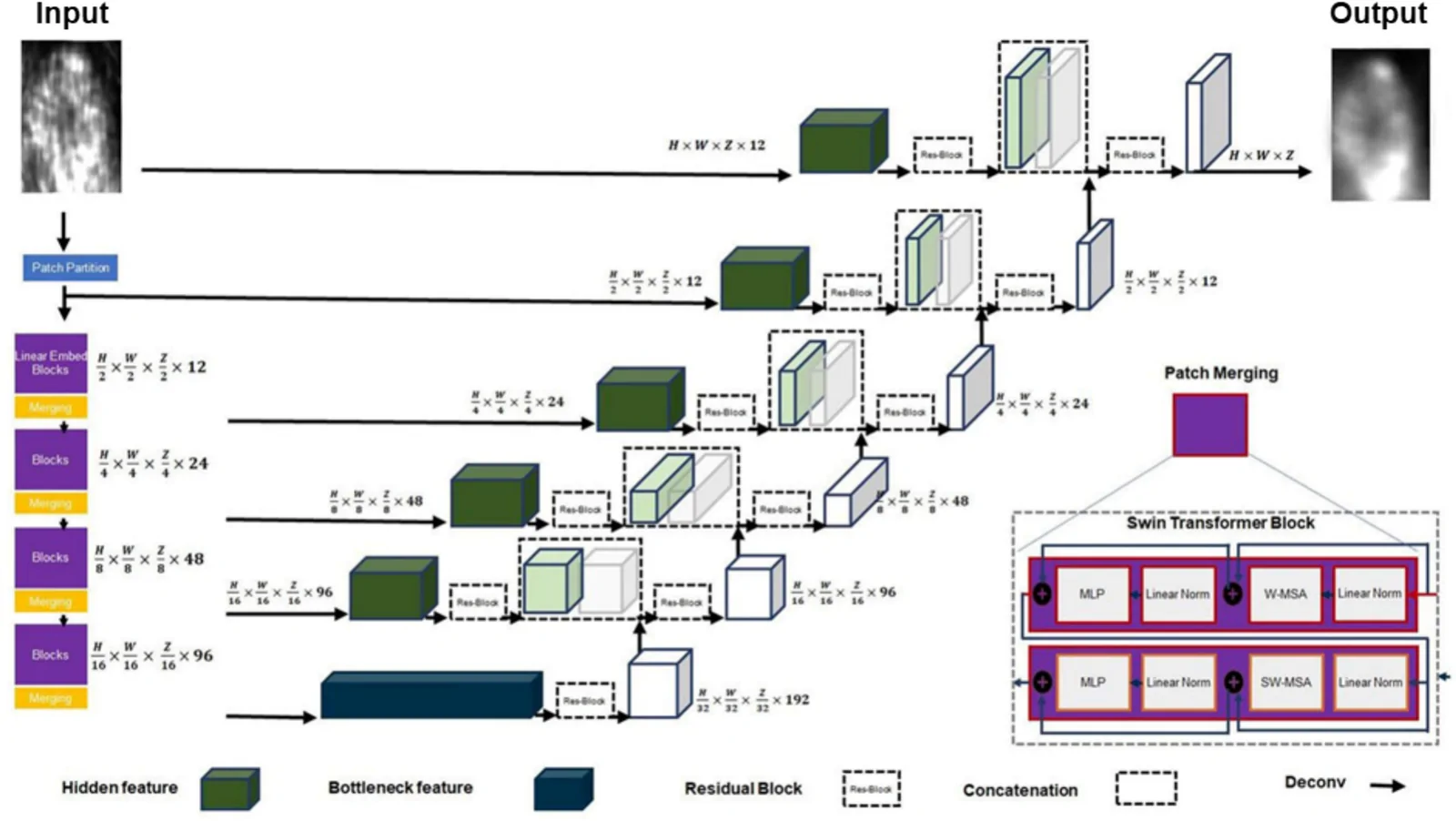
Overview of the Swin UNETR architecture. The Swin UNETR produces distinct patches from the input data and employs a patch partition layer to create windows of a defined size for self-attention computation. The encoded feature representations within the Swin transformer are subsequently transferred to a CNN-decoder via skip connections at different resolutions.

In this context, q, k, and *v* are elements of $R^{M^{2}\times d}$, representing the query, key, and value parameters. The quantity of patches in a window and the dimension of the query/key are denoted as $M^{2}$ and *d*, respectively. The architecture of SwinUNETR is depicted in Fig. 2.

The Swin UNETR model was trained to generate full-ring-equivalent PET images from dual-panel PET inputs using a supervised learning framework. The model was trained on 70% of the dataset with 10% used for validation, while 5-fold cross-validation was applied on the training data, and an independent 20% test set—kept completely unseen—was reserved for final evaluation. A dataset consisting of 51 paired image volumes was created, consisting of simulated dual-panel PET images as inputs and corresponding full-ring PET images as targets. Before training, both input and target images were preprocessed to ensure consistent voxel spacing, image dimensions, and intensity normalization. The model was trained on an NVIDIA GeForce RTX 2080 Ti GPU with 11 GB memory. Training required approximately 4 hours for each fold. Early stopping based on validation loss was used as the stopping criterion, with training halted if no improvement was observed over 20 consecutive epochs. Inference on a full 3D breast volume required about 30 seconds per case. During training, we used paired on the fly data augmentation on both input and target volumes, including small 3D rotations, translations, isotropic scaling, left right flips, and noise injection, to increase variability under limited sample size.

### Quantitative evaluation of simulated clinical images

The performance of DL-based methods was assessed using four quantitative metrics: the Structural Similarity Index Metric (SSIM), the Peak Signal-to-Noise Ratio (PSNR), and the Root Mean Square Error (RMSE), all based on the Standardized Uptake Value (SUV) All quantitative metrics (PSNR, SSIM, RMSE) were calculated on the entire 3D breast volumes after applying a breast mask, thereby excluding background voxels from the measurements. These metrics were computed using the following equations:

(6) RMSE=√MSE

(7) $$\mathrm{PSNR}=20.\log_{10}\left(MAX\right)-10.\log_{10}\left(MSE\right)$$

(8) $$SSIM\left(x,y\right)={\left[l\left(x,y\right)\right]}^{\alpha }.{\left[c\left(x,y\right)\right]}^{\beta }.{\left[s\left(x,y\right)\right]}^{\gamma }$$

In PSNR formula, MAX refers to the maximum possible intensity value of the image pixels, whereas in the SSIM formula, *x* and *y* represent the pixel intensity values at the corresponding coordinates in two images or image blocks being compared. The function *l(x,y)* evaluates luminance similarity, while *c(x,y)* assesses contrast similarity. The function *s(x,y)* measures structural similarity between the two images. The parameters α, β and γ denote the weighting exponents for the luminance, contrast, and structure components, respectively. These exponents are typically set to 1 to ensure equal contribution of each component to the final SSIM value. For PSNR calculation, the MAX value was defined as the maximum SUV within each individual reference (full-ring) image. This ensured that the dynamic range was image-specific and avoided reliance on an arbitrary theoretical maximum, thereby providing a consistent normalization across all cases. All quantitative metrics (PSNR, SSIM, RMSE) were calculated on the entire 3D breast volumes after applying a breast mask, thereby excluding background voxels from the measurements. A joint histogram analysis was also performed to depict the voxelwise correlation of the dual-panel images and Al quality-enhanced images by considering the cylindrical configuration as standard of reference.

To assess local structural preservation, SSIM maps were computed between the dual-panel images and the full-ring standard of reference, and between the DL-enhanced images and the full-ring standard of reference. These SSIM maps were displayed along with the corresponding bias maps to quantify both structural similarity and voxelwise intensity differences.

### Lesion-level analysis

A lesion-level quantitative analysis was performed to assess whether focal uptake was preserved by the DL model. Thirty cases containing breast lesions were randomly selected. Lesions were localized on the full-ring PET images and a spherical ROI was manually drawn around each lesion using 3D Slicer. The same spherical ROI was then applied to the dual-panel and DL enhanced images. For each lesion, SUV_mean_ was extracted. We then computed the average SUV_mean_ across the 30 lesions and the standard deviation across cases for each image type.

## Results

### Validation of Monte Carlo simulations

We assessed the spatial resolution and sensitivity at multiple locations within the FOV. Table 2 summarizes the obtained results. As expected, both spatial resolution and sensitivity deteriorate with increasing radial distance from the CFOV. The spatial resolution showed notable anisotropy across the x, y, and z directions, especially at 10 cm offset, where the radial FWHM increased to 4.2 mm compared to 3.2 mm at 1 cm. Sensitivity measurements also declined from 8.9 cps/kBq at the center to 5.8 cps/kBq at the 10 cm offset. These findings are consistent with the limited angular acceptance of the dual-panel design and highlight the need for spatially-aware analysis in system characterization.

**Table 2:** Spatial resolution FWHM (in mm) and sensitivity (in cps/kBq) for the dual-panel PET scanner at different radial positions within the CFOV.

Radial Offset(cm)	Radial (x)[mm]	Tangential (y) [mm]	Axial (z)[mm]	Sensitivity (cps/kBq)
1	3.2	3.1	3.3	8.9
5	3.6	3.4	3.7	7.5
10	4.2	3.8	4.1	5.8

Table 3 summarizes the comparison between the results of the experimental full-ring [51], the simulated full-ring Biograph mCT [40], a full-ring breast dedicated PET scanner [52] and our proposed scanner model.

**Table 3:** Comparison of the spatial resolution measured at 1 cm radial distance from the center of the field-of-view and sensitivity at the center of the field-of-view for partial breast PET configurations. ∅ denotes the diameter of the cylindrical PET scanner.

**Parameters**	Experimental Biograph mCT(∅=700 mm) [51]	Simulated Biograph mCT(∅=700 mm) [40]	Breast PET Full-ring (∅=230 mm) [52]	This Work Full-ring (∅=210 mm)	This WorkDual-panel(Distance= 210 mm)
Sensitivity (cps/kBq)	9.7	9.6	13.8	14.2	8.9
Spatial resolution (mm)	4.4	3.8	1.8	1.6	3.2

### Full-ring configurations

External benchmarks for the Biograph mCT at the CFOV are 9.7 cps/kBq from experiments [51], and 9.6 cps/kBq from simulation [40].We reproduced the simulated configuration and obtained comparable values, confirming the accuracy of our implementation. Our dedicated full-ring breast PET achieved 14.2 cps/kBq at the CFOV and 1.6 mm FWHM at 1 cm radial distance from the CFOV. The dual-panel configuration achieved 8.9 cps/kBq at the CFOV and 3.2 mm FWHM at 1 cm radial distance from the CFOV. Furthermore, the performance of our full-ring design is comparable to another dedicated breast PET scanner reported in [52], which achieved a sensitivity of 13.8 cps/kBq at the CFOV and a spatial resolution of 1.7 mm at 1 cm radial distance from the CFOV.

### Performance of DL-based methods

**Fig. 3:**
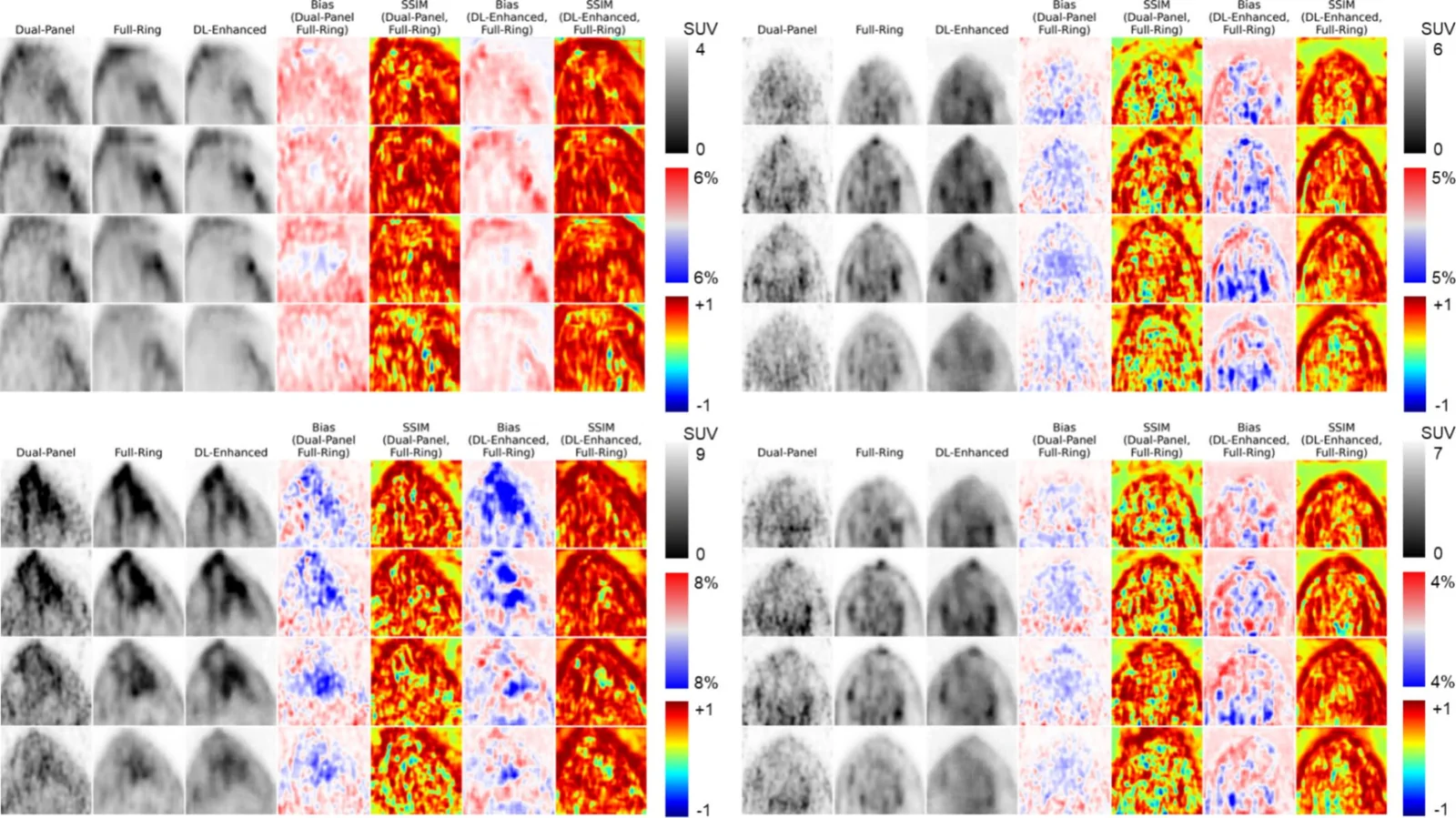
Representative slices from four cases showing the dual-panel, full-ring and DL-enhanced PET images, along with voxelwise bias maps and SSIM maps relative to the full-ring standard of reference. Bias maps display intensity differences, while SSIM maps depict local structural similarity. The DL-enhanced images show higher SSIM and reduced bias compared with the dual-panel images, indicating improved structural preservation.

The initial visual assessment demonstrated image quality improvement of the synthesized images (Fig. 3). The DL model successfully addressed challenges, such as limited angular coverage, and low image quality. It reconstructed images with improved quantitative fidelity and reduced artifacts compared with the dual-panel images.

Table 4 represents the outcomes of an analysis evaluating the quantitative metrics, including PSNR, SSIM, and RMSE, used to quantify improvements in image quality. There was a slight increase in PSNR from 24.81 to 25.47, indicating an enhancement of 2.65%. This increase signifies an improvement in signal fidelity and noise reduction. The SSIM increased from 0.61 to 0.78, showing a robust improvement of 26.43%. Additionally, the RMSE decreased from 0.05 to 0.04, indicating a reduction of 12.11%. To assess the statistical significance of these improvements, we performed Wilcoxon signed-rank tests across all 51 paired cases. The observed gains in PSNR, SSIM, and RMSE were statistically significant (p < 0.05), as summarized in Table 4.

**Table 4:** Image quality analysis of the synthesized PET images using image-derived metrics, including PSNR, SSIM and RMSE.

**Parameters**	**Full-ring vs. dual-panel**	**Full-ring vs. DL-enhanced**	Change **(%)**
PSNR	24.81	25.47	2.65 (p < 0.05)
SSIM	0.61	0.78	26.43 (p < 0.05)
RMSE	0.05	0.04	−12.11 (p < 0.05)

Fig. 3 shows representative slices of reconstructed images from the partial scanner, full-ring scanner, and predicted images using DL methods. Additionally, bias maps were calculated for the dual-panel scanner and quality enhanced images considering the clinical activity map as standard of reference.

The SSIM maps showed reduced structural similarity for the dual-panel images, particularly around regions with higher uptake and sharper gradients. The DL-enhanced images consistently produced higher SSIM values in the same areas, indicating improved preservation of local structure and texture. When viewed alongside the bias maps, the SSIM maps confirmed that the DL model reduced distortion present in the dual-panel reconstructions.

**Fig. 4:**
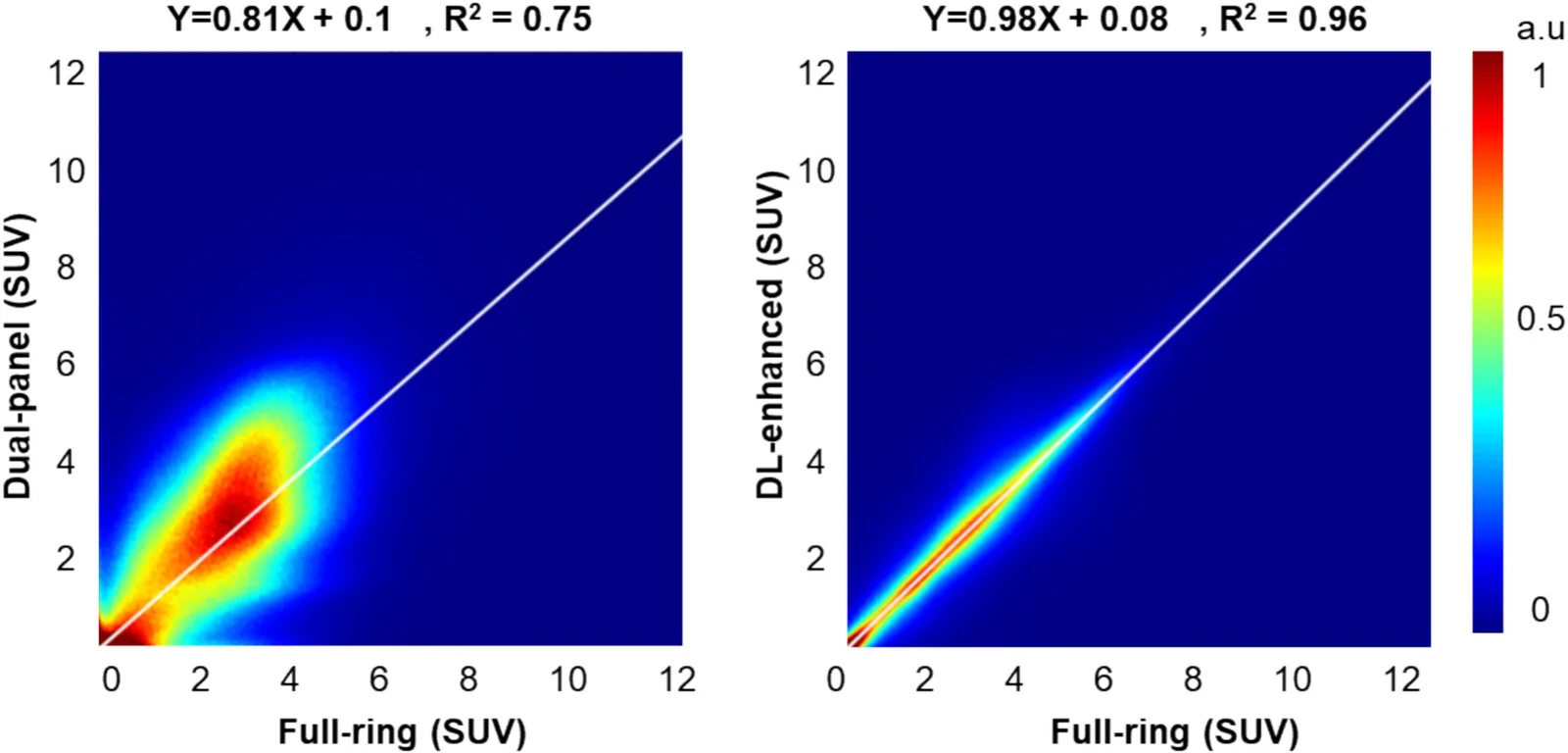
Joint voxelwise SUV histograms comparing dual-panel PET vs full-ring PET (left) and DL-enhanced PET vs full-ring PET (right). Color indicates normalized voxel density (arbitrary units, logarithmic scale). Both panels use the same color scale. The white line represents the identity line.

The voxel-wise correlation improved significantly after applying the DL model for image quality enhancement. Compared to dual-panel images, which showed a correlation of y=0.81x+01. with R^2^=0.75, the AI-enhanced images achieved a correlation of y=0.98x+0.08 with R^2^=0.96. These improvements in SSIM (+26%), PSNR, and voxel-wise correlation (R^2^ = 0.96) indicate enhanced quantitative fidelity and contrast consistency in AI-enhanced images, compared to dual-panel images. A dedicated clinical evaluation will be considered in future work. These results demonstrate that AI-enhanced images provide superior quantitative performance, highlighting their improved ability to preserve voxel-wise activity concentration correlations (Fig. 4).

### Lesion-level quantitative assessment

**Fig. 5:**
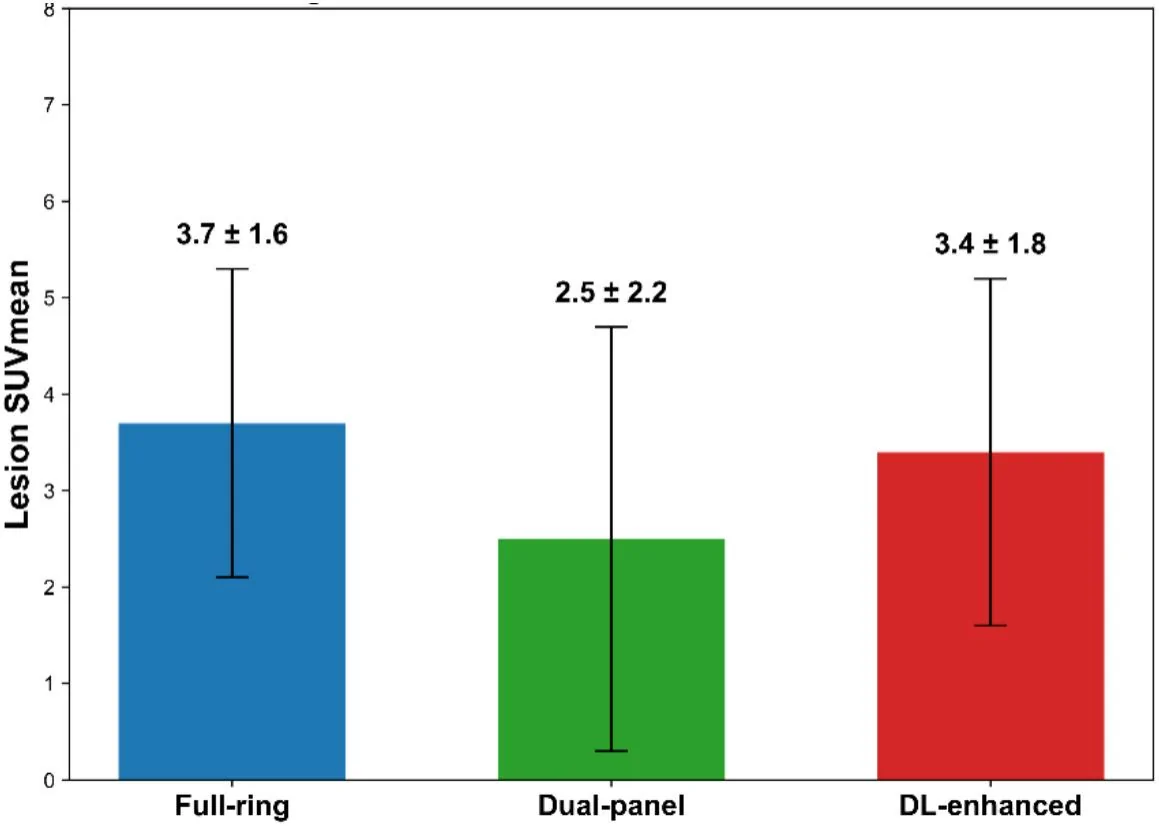
Average SUV_mean_ for 30 breast lesions extracted using spherical ROIs drawn on the full-ring PET images and propagated to the dual-panel and DL-enhanced images. Error bars represent the standard deviation across lesions. The dual-panel images underestimate lesion uptake. The DL-enhanced PET images reduce this bias and preserve lesion visibility.

Thirty breast lesions were analyzed using spherical ROIs. The full-ring PET images showed an average SUV_mean_ of 3.7 with a standard deviation of 1.6. The dual-panel images showed reduced uptake, with an average SUV_mean_ of 2.5 ± 2.2. The DL-enhanced images partially corrected this underestimation and reached an average SUV_mean_ of 3.4 ± 1.8. All lesions remained visible after DL enhancement (Fig. 5).

## Discussion

Partial-ring PET scanners have been proposed for dedicated applications, where limited angular coverage provides practical advantages such as improved surgical access [11], [15], [17], [53], [54], [55], [56].

In recent years, dedicated organ-specific PET scanners have shown success in achieving high sensitivity and spatial resolution. This success has sparked interest in these types of PET scanners, despite the cost and space requirements associated with having multiple scanners in one facility [57].

In particular, Huber et al. proposed a dedicated PET system that achieved a peak absolute sensitivity of 25.54 kcps MBq^−1^ at the CFOV and transverse spatial resolution of 4 mm FWHM at 1 cm from the CFOV [58]. The dual-panel acquisition in this work includes two discrete detector poses separated by a 90° step and shoot rotation, which increases angular sampling and improves isotropy compared with a fixed dual-panel geometry. This rotation reduces, but does not eliminate, the limitations of a partial system because the angular coverage remains incomplete relative to a full-ring and the sensitivity pattern remains highly nonuniform. Therefore, the deep learning model should be interpreted as an image restoration method operating on already improved two-pose data, not as a method that compensates for a purely missing angle acquisition. In practice, the model primarily addresses residual artifacts, spatially varying resolution and bias, and noise amplification that persist after iterative reconstruction of the two-pose dual-panel data and learns a mapping toward the full-ring reference under matched simulation and reconstruction settings. In this study, a partial system was designed for a dedicated breast PET scanner, and the images were restored using a DL approach. Metrics such as sensitivity, FWHM were calculated and compared with results reported in the literature, demonstrating good agreement with previous studies.

The sensitivity of the proposed PET scanner configuration is comparable to the breast dedicated PET developed by Emami et al. [52], with the spatial resolution at the center of the axial FOV being slightly better than the one in the reference above.

The dual-panel configuration achieved 8.9 cps/kBq at the CFOV and 3.2 mm FWHM at 1 cm radial offset. These values are comparable to dedicated breast PET designs reported in the literature, which show sensitivities around 7.2 cps per kBq and spatial resolution in the 2.5 to 4 mm range for breast-specific systems, and to full-ring breast systems near 13.8 cps per kBq with about 1.7 mm resolution. Our results therefore fall within the established performance envelope for clinical breast imaging systems and support feasibility for lesion depiction in a dedicated setting. Our findings are also in line with other dedicated breast PET systems recently reported. Samanta et al. used Monte Carlo simulations to compare a dedicated total breast PET with a clinical whole-body PET, reporting a sensitivity of ∼8.5 kcps MBq^−1^ and spatial resolution of ∼4 mm FWHM, values similar to those obtained in our system [59]. Likewise, Morimoto-Ishikawa et al. evaluated a high-resolution TOF PET system dedicated to head and breast, achieving ∼7.9 kcps MBq^−1^ sensitivity and ∼4.1 mm spatial resolution [60]. These results confirm that the performance of our dual-panel system falls within the expected range of breast-dedicated PET designs. In addition, AI enhancement improved quantitative agreement with the full-ring reference, including a 26 percent increase in SSIM and a 12 percent reduction in RMSE, which mitigates limited-angle artifacts and supports contrast preservation. Together, these points justify that the measured sensitivity and resolution are sufficient for dedicated breast imaging use, while we note that prospective reader and lesion-level studies remain necessary to confirm clinical impact.

Hardware and reconstruction-based strategies, such as DOI encoding, PSF modeling, and TOF can reduce parallax errors and improve the accuracy of each individual line of response. These methods can be highly effective when implemented with appropriate detector technology. Our approach operates at the image level and reduces residual artifacts and bias caused by incomplete angular sampling and nonuniform sensitivity, even with the two-pose acquisition. It can also reduce residual bias and restore global structure even when TOF, PSF modeling, or DOI correction are present. In settings where such advanced hardware is not available, the method provides a practical complement that helps mitigate geometric and angular limitations.

Our DL-based restoration approach improved image quality in the dual-panel PET system, achieving superior quantitative performance and preserving voxel-wise activity concentration, thereby mitigating limitations inherent in partial-ring PET designs. Such approaches, including DOI encoding, PSF modeling, and TOF implementation, have been proposed to mitigate artifacts and improve image quality [61], [62], [63], [64], [65], [66].

The high R^2^ value may largely reflect agreement in broad uniform regions and can miss small but important lesion differences. For this reason, we separately calculated the lesion level bias (Fig. 5), which demonstrates how lesion uptake is specifically affected in the dual-panel and DL-enhanced images. In future work, we will evaluate lesion detectability and conduct more rigorous clinical assessment and quantitative analysis.

Applying the DL-based method to different body sections should be feasible, although more challenging due to the diversity in tissue types and shapes. Ensuring accurate and robust predictions requires training the DL-based method with relevant data that matches the conditions of the deployment data, such as varying activity levels, diseases, and PET tracers [67]. Additionally, exploring various advanced neural network architectures, including generative adversarial networks (GANs), should be considered to improve performance [68]. Using a small sample size for training the model was another limitation of the present study, which we tried to minimize through data augmentation. Yet, this can be addressed by enlarging the dataset in future works.

This study modeled only true coincidences and did not include scatter or random events. Incorporating these additional components would make the simulations more realistic and would increase image noise in both the dual-panel and the full-ring configurations. This limitation does not affect the comparative design of the study but reduces the degree of realism of the generated images. Future work will extend the simulation framework to include full scatter and random modeling to better reflect clinical acquisition conditions. This study investigated the performance of the DL-based method for restoring and improving images obtained from breast PET partial detector systems. However, to ensure the application of DL for restoring degraded images, further training using a larger and more diverse dataset, along with exploration of different neural network architectures, could further improve the model’s generalization and performance. Adversarial training can encourage sharper structural detail and better preservation of local contrast compared with purely supervised losses. GANs can help counteract the smoothing tendencies of voxelwise loss functions and may recover fine textures or lesion boundaries that are challenging in limited-view acquisitions, although they also require careful control to avoid hallucinated structures.

## Conclusion

In conclusion, this study presented a comprehensive simulation framework to evaluate deep learning-based image enhancement for a dedicated dual-panel breast PET scanner. The dual-panel acquisition used a two-pose step and shoot 90° rotation to increase angular sampling. Hence, the deep learning results should be interpreted as a methodology enabling to reduce residual artifacts, noise, and quantitative bias remaining after iterative reconstruction of the two-pose data, rather than compensating for a purely single-pose missing angle geometry. Overall, the proposed approach improved agreement with the full-ring reference and better-preserved voxel-wise activity concentration.

## CRediT authorship contribution statement

**Hamid Nezampour:** Writing – original draft, Validation, Software, Methodology, Investigation, Formal analysis, Data curation, Conceptualization. **Mehdi Amini:** Writing – review & editing, Visualization, Validation, Methodology, Investigation, Conceptualization. **Amirhossein Sanaat:** Writing – review & editing, Visualization, Supervision, Software, Formal analysis, Data curation, Conceptualization. **Habib Zaidi:** Writing – review & editing, Supervision, Project administration, Methodology, Investigation, Funding acquisition, Conceptualization.

## Declaration of competing interest

The authors declare that they have no known competing financial interests or personal relationships that could have appeared to influence the work reported in this paper.
